# Bioavailability and health risk assessment of potentially toxic elements in popcorn kernel from sandy loam Ferric Luvisol amended with municipal solid waste compost

**DOI:** 10.1007/s10653-021-01020-y

**Published:** 2021-07-02

**Authors:** Oyeyemi A. Dada, Funso R. Kutu

**Affiliations:** 1 Food Security and Safety Niche Area Research Group, North-West University, Mafikeng Campus, Mmabatho, 2735 South Africa; 2 School of Agricultural Sciences, University of Mpumalanga, Mbombela, South Africa

**Keywords:** Popcorn kernels, Toxic elements, Compost utilization, Health risk, Metal(loid) pollutants

## Abstract

Application of municipal solid waste compost (MSWC) to marginal soil enhances crop growth but could also serve as source of pollutants into agroecosystem. There is scanty report on bioavailability of potentially toxic element (PTE) and the health risk of consuming popcorn kernel harvested from field fertilized with MSWC. Field trial was carried out in 2017–2019 to evaluate bioavailability of PTEs in kernel of popcorn harvested from field fertilized with MSWC. The trial was conducted at the experimental field of North-West University, Mafikeng campus South Africa. The treatments comprised three rates of MSWC including 0 t/ha (unamended, control), 4 t/ha and 8 t/ha arranged in randomized complete block design and replicated four times. One seed of popcorn was sown at 20 × 70 cm spacing per hole of 3 cm depth in a 6 × 4.2 m plot size. Growth and yield data were collected at maturity. Ears were harvested at maturity and the kernels were dried to 12% moisture content. Air-dried kernels (50 g) samples were collected and analyzed for essential mineral nutrient and some heavy metal(loid)s using ICP-MS. Measured concentrations of these heavy metal(loid)s were then used to calculate the health risk for adults and children. The results showed that uptake concentration was in the order K^+^ > HPO_4_^2−^ > Mg^+2^ > Ca^2+^ > Fe^2+^ > Cr^6+^ > Zn^2+^ > Mn^2+^  > Cu^2+^(mg/kg). Uptake concentration of metalloids: Al and Pd was significantly higher in the unamended. Bioavailability of PTE was highest in unamended plots. The average daily intake of the PTEs was within the recommended permissible level. The risk index value for oral pathway was < 1 for both adult and children population. Amending Ferric Luvisol with 80 t/ha MSWC enhanced popcorn growth and, concentration of accumulated PTEs in kernels at this rate, cannot pose health risk to both adult and children population.

## Introduction

Popcorn (*Zea mays everta* L.), a low-fat whole-grain snack is a pleasant delight in many homes, parks and recreational centers. The popped kernel is a rich source of vitamins, insoluble fiber and antioxidants, especially phenolic acids (Coco & Vinson, 2019). Its nutritional value is far greater than many commonly consumed grain or potato based snacks. According to Nguyen et al. (2012), consumption of popcorn as a chow is more beneficial in reducing cardiovascular incidence in humans. The kernel is low in calories and rich nutrient source as grub than veggies, fruits and some other whole grain like wheat (de Graaf, 2006, Grandjean, 2008). Popcorn belongs to the family poaceae and performs excellently well on well drain fertile soil.

Plants do not discriminate which nutrient to uptake or evade in the soil. Availability of mineral nutrients in the rhizospheres, its uptake by plants and translocation to other plant parts are not mutually exclusive (Li et al., 2016). It therefore implies that availability of ions from a source into a sink depends on what mineral was taken up from the soil environment. In actual fact, plants uptake dissolved ions (macro, micro and beneficial elements) in solution form for biosynthesis of many essential compounds. The absorbed ions are expected to confer benefits on the plant but could become harmful when it attains certain concentration plane. For instance, Kovács et al. (2009) found that Pb^2+^, Cd^2+^As^5+^ and Ti^+^ at 5 × 10^–8^ concentration can effect stimulatory influence on certain metabolic processes in cereal crops. On the other hand, accumulation of some of these minerals ions beyond certain concentration may become toxic to plants (Fosu-Mensah et al., 2017). Equally, Ogiyama et al. (2005) showed that some heavy metals like Cu, Cr and Zn are essential trace nutrients and may become toxic at high concentration. For example, Ogiyama et al. (2005) and Gupta et al. (2016) affirmed that plants tolerance to ions concentration varies among different species. Some plants, classified as accumulators adsorbed and translocate ions at hazardous level as photoassimilate into kernels, leaves, grains and fruits (Boyd, 1998) while some are intolerant of high concentration of metal ions. The uptake, distribution and concentration of ions in kernel of popcorn in field fertilized with composted organic waste have not been thoroughly studied.

Appropriate disposal of wastes in many municipalities is germane to prevent different forms of environmental pollutions. In this wise, composting, a scientific means of waste disposal management is an age long practice employed in recycling nutrients in agricultural systems. It is a major mean usually deployed for waste reduction, reutilization of some organic and inorganic materials and recycling of essential nutrients. Wastes generated and gathered from different parts of municipalities can be reduced to 40–50% through composting and the output can be used as soil amendment (Singh et al., 2010). Composting is an important curing process that helps to destroy pathogens in waste metabolically during the heat or thermophilic phase of the transformation process. This makes compost a good source of organic and inorganic plant nutrients (Couth & Trois, 2012). In spite of its waste management and nutrient recycling strategy, compost could also serve as the route through which potentially toxic elements and metalloid pollutants enter the food chain.

In many agricultural fields, soil are supplied with plant minerals from diverse sources such as mineral fertilizers or organic sources like farmyard manure, plant residue and compost of various origins (Khan et al., 2018). Compost derived from municipal solid waste is a major source of organic soil amendment in many fields. However, application of compost, farm yard manure, and biochar as fertilizer may be a source of metal pollutants into agroecosystem. Some of these pollutants and hazardous substances enter food chain through anthropogenic and natural pathways (Sarwar et al., 2017). Excessive uptake of hazardous metals from organic waste amended soil have been reported in rice (Hseu et al., 2010), vegetables (Li et al., 2017) and wheat (Guo et al., 2018). Nevertheless, research on concentration and implication of metal pollutants constituents in grains of popcorn plant grown with composted recycled municipal waste materials have not been studied so far.

Optimizing nutrient recycling via compost utilization is one side of a coin, it is another plane to ensure food safety by preventing consumption of hazardous substances that are takening up in field amended with compost derived from municipal wastes (Khan et al., 2018). Evidences abound of the benefits associated with using municipal solid waste compost (MSWC) (Huang et al., 2016; Venegas et al., 2016). Availability and uptake of heavy metals in soil is a function of prevailing anthropogenic (cultural system) and soil or edaphic characteristics (Thielecke & Nugent, 2018). Application of MSWC to soil may introduce contaminants into the uncontaminated field as reported by Farrell et al. (2010). There, is however, little information on transfer of potentially toxic elements including metalloid pollutants into the kernels of popcorn on field amended with MSWC.

In order to ensure kernel quality and consumers’ safety, it is important to ascertain that bio-concentration of heavy metals and metalloids in kernel of popcorn grown on Ferric Luvisol amended with MSWC are within the global acceptable limits. In contrast, uptake and bioavailability of metal and metalloid pollutants in rhizosphere may be altered by compost application according to Pérez-de-Mora et al. (2006). However, information on bioavailability and health risk of metal pollutants in kernel of popcorn in Ferric Luvisol amended with MSWC is sketchy particularly in semi-Arid region which have high potential for popcorn cultivation. This information is important to assess heavy metal pollutant concentrations in kernel of popcorn to ensure food safety, prevent environmental and public health risks. Hence, this study investigated bio-concentration of some toxic metals and metalloids in kernel of popcorn grown on Ferric Luvisol amended with MSWC and the health risk implications. This will be the first report where records of metals and metalloids in grain or kernel of popcorn were determined. The information will be useful for policy makers on health and biowaste management.

## Materials and methods

### Description of the study area

**Fig. 1:**
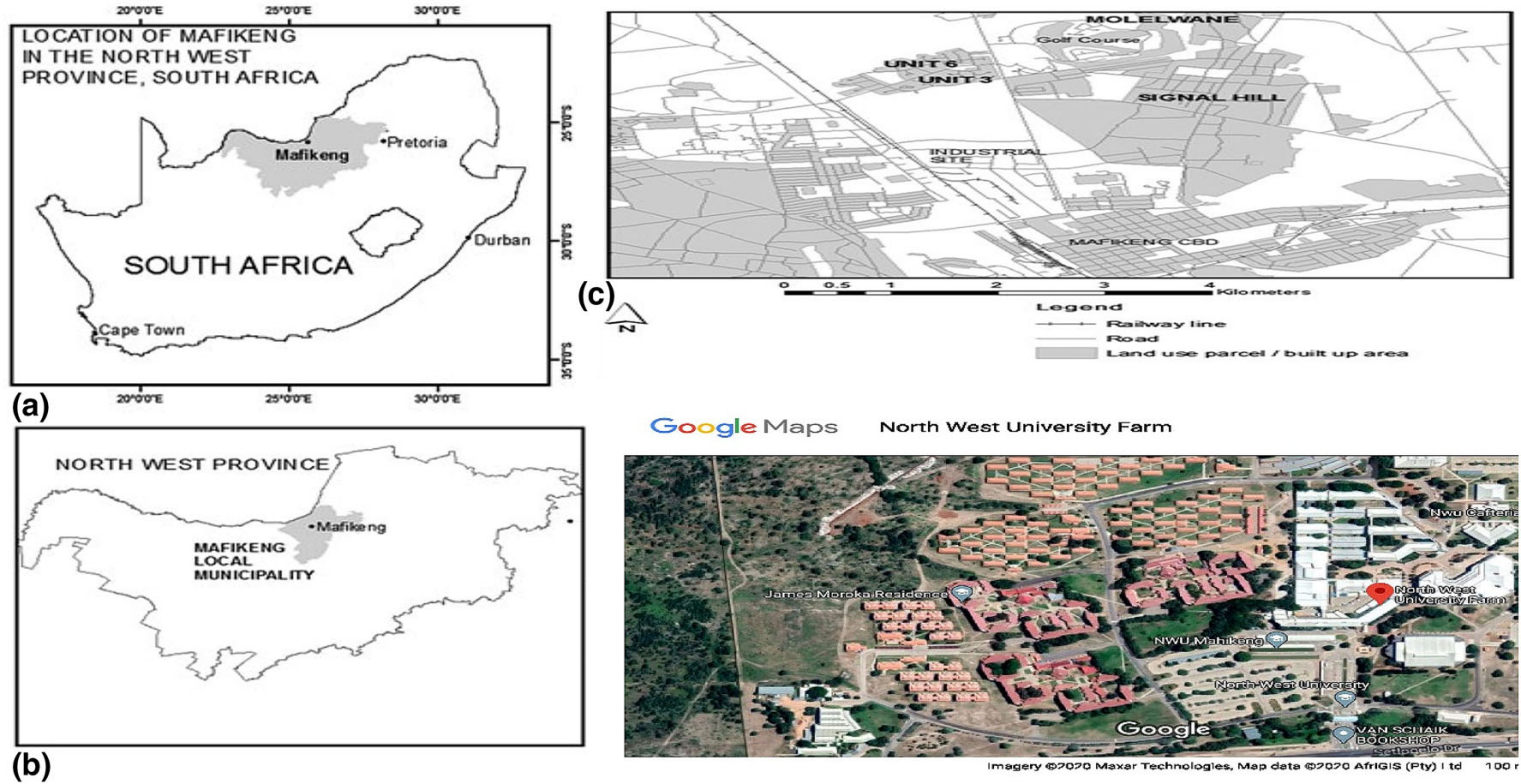
Map of South Africa showing. **a** Mafikeng local municipality in North-West Province, **b** Molelwane, **c** the study site location, **d** North-West University Farm (Munyati & Motholo, 2014)

The study area is located in North-West University farm, Molelwane in North-West Province of South Africa (25°49′39″S, 25°36′3″0 E, 1280 m above the sea level.) during 2017/2018 and 2018/2019 summer planting seasons. The summary of atmospheric conditions of the study location during the field trials showed that the mean temperature, rainfall and relative humidity were 20.2 °C, 84.13 mm and 60.2% in 2017/2018 and 23.85 °C 137.73 mm and 58.5% in 2018/2019 seasons, respectively. Equally, The study location is shown in Fig. 1. The field had been under intensive cultivation for more than a decade. The field is reserved for experimental purposes on major arable crops like maize (*Zea mays)*, cowpea (*Vigna unguiculata* L.), sorghum (*Sorghum bicolor)* and dry bean (*Phaseolus vulgaris*). In the previous three summer seasons (2013/14, 2014/15 and 2015/16) the portion of the field used for this study was cultivated with maize, cowpea and maize in succession. During each planting period diverse form of agrochemicals such as fertilizers especially, NPK and pesticides were applied as cultural management practices for these crops. Also, many researches that involved evaluation of different farm yard manures on performance of above listed arable crops had been conducted on this field in previous seasons. Irrigation water was usually source from public water supply to supplement moisture supplied by rain.

## Composting, compost and soil analyses

The municipal solid waste compost (MSWC) used for this investigation was prepared at the composting shed of North-West University, Mafikeng Campus. The MSWC was produced from combination of sorted MSW incorporated with poultry (PM), cattle (CM), sheep (SM) manures and wood shavings (WS) at a ratio of 4.5:4.5:2:3:1 (MSW:PM:CM:SM:WS) on dry weight (kg) basis.

The PM, CM and SM were collected from pens of the respective livestock at the Animal Science unit of NWU experimental farm. The WS was collected from industrial area in Mafikeng and MSW was from a waste dumping site in Lichtenburg, which is approximately 72 km away from the composting site. Thermophilic anaerobic heap method was used for the decomposition process. Each Layer of the materials comprising 321 kg/t MSW, 321 kg/t PM, 143 kg/t SM and 214 kg/t WS were made on flat floor laid with 100 mm thick polythene sheet to form a heap (1 ton) under a protected shed. The heaps were turned and moderately sprinkled with water bi-weekly until maturity (16 weeks after preparation).

The final compost produced was chemically characterized. Analyses of samples were carried out in the laboratory following standard procedures described by Landon (2014) for soil and Lasaridi and Stentiford (1998) for compost. The physical and chemical characteristics of the soil used for the trial as well as chemical constituents of the cured compost are presented in Table 1. The results indicated that the soil is marginal as the primary mineral nutrient contents like N, P and K are below the quantity adequate for optimum growth and development of popcorn plant.

**Table 1:** Physico-chemical characteristics of soil and cured compost used for the study

Characteristics	Soil	Compost	EU	South Africa*
Sand %	81	na	na	na
Silt %	3	na	na	na
Clay %	16	na	na	na
Textural class	Sandy loam	na	na	na
pH (H_2_0)	8.2	7.8	na	na
pH (KCl)	7.1	7.4	na	na
Electrical conductivity (µs)	78.6	70.88	na	na
Total C (g/kg)	18.0	134	na	na
Total N (g/kg)	0.5	35,559.2	na	na
Na (g/kg)	0.02	3.3	na	na
Mg (g/kg)	0.3	7.2	na	na
Al (g/kg)	33.26	16.2	na	na
P (Bray1) (g/kg)	0.1	1.4	na	na
K (g/kg)	0.3	10.2	na	na
Ca (g/kg)	0.7	119.5	na	na
Mn (mg/kg)	39.8	1288	na	740
Fe (mg/kg)	7.9	1172	na	na
Co (mg/kg)	nd	1.2	300	300
Cr (mg/kg)	nd	11.6	150	6.5
Ni (mg/kg)	14.7	16.2	75	91
Cu (mg/kg)	0.7	54.4	140	16
Zn (mg/kg)	11.8	11.6	300	240
As (mg/kg)	0.2	0.8	20	5.8
Mo (mg/kg)	nd	31.6	na	na
Pb (mg/kg)	0.19	3596.1	300	20
Cd (mg/kg)	0.06	2312	3	7.5

*nd* not determined, *na* not available

*Maximum allowable limit of concentration of heavy metals (mg/kg) for EU and South Africa (Commission, 2011; Water & Affairs, 2014; Kamunda et al., 2016a)

## Field layout and experimental design

The treatments comprised three rates of MSWC including 0 t/ha (Control), 4 t/ha and 8 t/ha which were arranged in randomized complete block design and replicated four times. The different compost rates were applied in dry weight, based on nitrogen content. The MSWC rates evaluated were based on previous study conducted on similar environment (Oworu et al., 2010, Kutu et al., 2018). The compost application was done each in December of 2017 and 2018. This was done to allow the applied materials mineralized for four weeks before planting in January of 2018 and 2019, respectively. One seed of popcorn was sown at 20 × 70 cm spacing per hole of 3 cm depth in a 6 × 4.2 m plot size resulting in 95,238 plants/ha. The field was managed appropriately following standard agronomic practices until maturity. Growth and yield data were collected at maturity.

## Determination of concentration of potentially toxic elements and metalloids uptake in popcorn kernels

Ears were harvested at maturity and kernels were obtained per treatment. The ears were spread on a tarpaulin under a processing shed to air dry until needed for further analyses. The kernels were air-dried until it attained 12% moisture content under the shed. The moisture content was determined with a handheld Dickey-John grain moisture tester (Model, MiniGAC1SG4).

## Sample digestion with high-performance microwave digestion system

Air-dried kernels (50 g) were obtained per treatment and kept in zip lock plastic bags for heavy metals and metalloids determination using microwave digestion method described by Mataveli et al. (2016). The kernels were microwave digested (Ethos UP, Magna Analytical) with EPA3051A method. The sample was weighed (200 mg) and placed in a Teflon tube. A volume of 9 ml 65% nitric acid (HNO_3_) and 3 ml 32% hydro chloric acid (HCl) was added to the tube, capped and placed in a high-performance microwave digestion system (Milestone, Ethos UP, Maxi 44). A period of 20 min allowed the system to reach 1800 MW at a temperature of 200 °C which was maintained for 15 min. After cooling, the sample was brought up to a final volume of 50 ml and analyzed using an ICP-MS (Agilent 7500 series).

## Trace metal analysis using ICP-MS

Agilent Inductively Coupled Plasma-Mass Spectrometry (ICP-MS) model PerkinElmer NexION 300 ICP-MS (PerkinElmer, Waltham, MA, USA) was used to obtain the concentrations of analytes in the digested kernel samples. Primarily, ICP-MS is used to determine metals in various matrices at trace level. First, all samples were digested in HNO_3_ according to EPA 3050b. Subsequently samples were analyzed on an Agilent 7500 CE ICP-MS fitted with CRC (Collision Reaction Cell) technology for interference removal. Sample introduction was achieved via a Micromist-type nebulizer (reducing matrix interferences and for a more robust plasma) and standard quarts spray chamber. The instrument was optimized using a solution containing Li, Y, Ce and Tl (1 ppb) for standard low-oxide/low interference levels (≤ 1.5%) while maintaining high sensitivity across the mass range. Typical instrumental conditions are as indicated in Table 2. To obtain quantitative results, the instrument was calibrated using ULTRASPEC® certified custom mixed multi-element stock standard (De Bruyn Spectroscopic Solutions, South Africa) solutions containing all the elements of interest. Quality Control Standards are employed to assure the correct QC criteria are met.

**Table 2:** Typical ICP-MS instrument conditions for analyzing trace metals

Parameter	Value
Forward power	1550 W
Plasma gas flow	15 L/min
Nebulizer gas flow	1.2 L/min
Sampling depth	8 mm
Spray camber temp	2 °C

## Determination of metal mobilization ratio

Metal mobilization ratio was calculated to determine relative translocation of metals from soil to kernel of the popcorn. The soil-transfer constant was evaluated as concentration of a heavy metal/metalloids in kernel of popcorn (dry weight) relative heavy metal concentration in the soil treated with or without MSWC as follows:

1 $${\text{TC}} = \frac{{C_{{{\text{plant}}}} }}{{C_{{{\text{soil}}}} }},$$

where TC is transfer coefficient (mg/kg), *C*_plant_ is heavy metal(loid)s concentration in popcorn kernels tissue (mg), and *C*_soil_ is metal(loid)s concentration in soil (kg dry soil). Our plant tissue sampling for laboratory analysis was limited to grain while data on the stover was limited to dry weight accumulation.

## Health risk assessment

Human health risk was assessed by determining the pathway of human exposure to heavy metals. In this study, we employed the health risk assessment model generated by United State Environmental Protection Agency (U.S. EPA) to evaluate human health risk of metal(loid)s for adults and children.

## Daily intake of metals

The daily intake of metals was determined using oral intake of crop exposure pathway described by Khan et al. (2008) and Liang et al. (2017). The equation for estimating daily intake of metal(loid)s is given as follows:

2 $${\text{ADI}}_{{{\text{crop}}}} {\text{ }} = \frac{{C_{{{\text{crop}}}} \times {\text{IR}}_{{{\text{crop}}}} \times {\text{CF}} \times {\text{EF}} \times {\text{ED}}}}{{{\text{BW}} \times {\text{AT}}}},$$

where ADI is the average daily intake of heavy metal(loid)s ingested from kernel sample in mg/kg/day, *C* is concentration of heavy metal(loid)s in mg/kg for popcorn. IR in mg/day is the ingestion rate, EF in days/year is the exposure frequency, ED is the exposure duration in years, BW is the body weight of the exposed individual in kg, AT is the time period over which the dose is averaged in days.

The average daily intakes for adults and children were considered to be 0.30 and 0.25 kg person^−1^ day^−1^, respectively, while the average adult and child body weights were considered to be 70 and 15 kg, respectively, as used in previous studies (Kamunda et al., 2016a; Kusin et al., 2018). Other oral exposure factors for determining health risk index are shown in Table 3.

**Table 3:** Oral exposure parameters used for the health risk assessment through different exposure pathways for soil

Parameters	Unit	Child	Adult	References
Ingestion rate (IR)	mg/kg	200	100	Kamunda et al. (2016b), Kusin et al. (2018)
Exposure frequency (EF)	days/year	350	350	Kamunda et al. (2016b), Kusin et al. (2018)
Exposure duration (ED)	year	6	30	Kamunda et al. (2016b), Kusin et al. (2018)
Conversion factor (CF)	kg/mg	10^–6^	10^–6^	Kamunda et al. (2016b), Kusin et al. (2018)
Body weight (BW)	kg	15	70	Kamunda et al. (2016b), Kusin et al. (2018)
Average time (AT)				
For carcinogens	days	365 × 70	365 × 70	Kamunda et al. (2016b), Kusin et al. (2018)
For non-carcinogens	days	365 × ED	365 × ED	

## Non-carcinogenic risk assessment

Non-carcinogenic hazards evaluated hazard quotient which is a unitless number that is expressed as the probability of an individual suffering from an adverse effect. The Health Risk Index (HRI) through the consumption of metal(loid)s contaminated popcorn samples was assessed based on the food chain and the reference oral dose (RfD) for each of As, Cr, Cu, Fe, Hg, Mn, Ni, Pb, Co and Zn. The health risk index (HRI) otherwise known as Hazard Quotient (HQ) was calculated using the formula below:

3 $${\text{HRI}} = {\text{HQ}} = \frac{{{\text{DIM}}}}{{{\text{RfD}}}},$$

where DIM = Daily intake of metal; RfD = Oral Reference. The HRI < 1 means the exposed population is assumed to be safe.

For *n* number of heavy metals, the non-carcinogenic effect to the population is as a result of the summation of all the HQs due to individual heavy metals. This is considered to be another term called the Hazard Index (HI) as described by Means (1989). The mathematical representation of HI is shown below as follows:

4 $${\text{HI}} = \mathop \sum \limits_{{k = 1}}^{n} {\text{HQk}} = \mathop \sum \limits_{{n = 1}}^{n} \frac{{{\text{DIMk}}}}{{{\text{RfDk}}}},$$

where HQk, DIMk and RfDk are values of heavy metal or metalloids k. When the HI value is less than one, the exposed population is unlikely to experience adverse health effects. However, if the HI value exceeds one, then there may be concern for potential non-carcinogenic effects (Means, 1989).

## Data processing and statistical analysis

Analysis of variance (ANOVA) as well as descriptive statistics of all the data collected was done using General Linear Model (GLM) of statistical Analysis System (SAS) software, version 9.3.

## Results and discussion

### Growth, yield and mineral nutrient uptake response of popcorn to compost application

The compost used is rich in essential plant nutrients, alkaline in nature with substantial amount of organic carbon which was 86% higher than the organic carbon in the soil. Surprisingly, the concentration of Lead (Pb) in the compost was outrageously high beyond the allowable limits for all countries (Table 1). The extremely high Pb concentration could be linked to the source of the raw materials used to prepare the compost. Equally, other heavy metals and metalloids were found in the compost but none was above the permissible level of soils in South Africa (Herselman et al., 2012; Kamunda et al., 2016a) and many other countries (Commission, 2011).

The concentration of toxic metals found in the soil prior to application of MSWC was in trace quantity below the risk threshold. It was unlikely that the major source of these trace quantity was probably through agrochemical application as well as other anthropogenic activities. As reported above, the field where the trial was carried out had been used for various researches involving application of soil amendments like farmyard manure, organo-mineral fertilizers, pesticides and many others. Similar observation had been reported by Hooda (2010) and Lim et al. (2016) in arable field where minutes quantity of metals where found in untreated soil samples.

Application of MSWC affected growth of popcorn significantly. Growth and yield of popcorn improved tremendously in field fertilized with MSWC relative to the unfertilized plots (Table 4). The number of leaves formed, height of the plant and total biomass were better significantly in compost amended plots over and above plants that depended on native soil nutrients. The performance of the crop in terms of growth and yield, increased steadily with increase in the rate of the applied compost. Weight of 100 seeds (20.10 g) and grain yield (8.39 t/ha) were significantly affected by soil amendment as these yield parameters were higher significantly in plots fertilized with 8 t/ha. This confirmed the relevance of organic fertilizers in enhancing fertility status of soil which ultimately improved performance and yield of crops. Utilization of compost as soil amendment for improving soil fertility in arable fields toward improving the performance of crop had been well documented. It had been shown that compost is a veritable source of essential mineral nutrients for plant uptake. Kirchmann et al. (2009), Jin et al. (2017), Thomas et al. (2019) have shown that MSWC as organic fertilizer, is a good alternative to fossil based fertilizer for enhancing plant growth and development. Views of Sayara et al. (2020) and Kranz et al. (2020) agreed with our submission that composting is a vital mean of recycling biowastes and their usefulness in agriculture by enhancing soil characteristics and improving crop yield.

**Table 4:** Growth and yield of popcorn as influenced by different rates of municipal solid wastes compost

MSWC Rate (t/ha)	Number of leaves	Plant height (cm)	Leaf area index	Total biomass (g)	Weight of 100 seeds (g)	Grain yield (t/ha)
0 (Control)	10.94	176.98	60.34	169.21	17.83	5.34
4	10.92	182.97	61.92	212.58	17.56	6.48
8	11.83	190.08	70.60	274.47	20.10	8.39
Mean	11.23	183.34	64.28	218.42	18.49	6.74
SEM ( ±)	0.42	6.74	3.87	23.89	0.53	0.41
LSD (*p* ≤ 0.05)	1.73	27.11	17.29	106.62	2.53	1.82

*MSWC* Municipal solid waste compost, *SEM* Standard error of means, *LSD* Least significant difference at *p* ≤ 0.05

The uptake concentration of macro and trace mineral nutrients into the kernel harvested from amended field decreased with increase in the quantity of the applied MSWC. There was no appreciable difference in the concentration of micro and macro nutrients assimilated into the popcorn kernel obtained in plots supplied with 4 or 8 t/ha MSWC (Table 5). This suggests that mineral nutrients released by MSWC was somewhat slower compared to uptake in the control plots. Oftentimes, nutrients in organic fertilizers are organically bound and hence their release does not always tally with crop requirement (Kirchmann et al., 2009). This may be related to the organic matter content of the MSWC which probably encouraged bounding of some of these minerals thereby reducing the rate at which they become solubilized and made available for uptake and partitioned into the kernel. The possibilities that some soil microbiota used some of the minerals in the compost for their metabolism may not be farfetched. This may explain the reason why the mineral nutrient concentrations in the grain of popcorn were inversely proportional to the quantity of the applied MSWC relative to the unamended plots.

**Table 5:** Uptake concentration (mg/kg) of essential plant nutrients in popcorn kernel

MSWC Rate (t/ha)	K	P	Mg	Ca	Cu	Fe	Mn	Mo	Zn	Na	Cr
0 (Control)	3640.0	3280.0	1706.0	236.60	3.93	155.20	9.74	1.06	31.68	29.34	47.90
4	2909.0	2174.3	1072.9	128.57	2.58	100.02	6.59	1.06	19.31	11.71	45.18
8	2610.0	1966.0	1047.6	114.73	2.61	112.22	7.18	1.04	17.12	11.26	38.36
Mean	3053.00	2473.44	1275.52	159.97	3.04	122.48	7.84	1.05	22.70	17.44	48.81
SEM ( ±)	186.43	229.89	116.61	22.26	0.25	8.87	0.52	0.04	2.41	3.04	1.96
LSD (*p* ≤ 0.05)	611.91	642.53	280.57	89.05	0.67	21.15	1.49	0.38	6.34	5.86	9.02

*MSWC* Municipal solid waste compost, *SEM* Standard error of means, *LSD* Least significant difference at *p* ≤ 0.05

The pattern of uptake concentration may be linked to the nature of the soil. Duarte et al. (2019) have reported that grain nutrient content can be affected by soil characteristics especially the organic matter richness. Also, the pH of the soil had influence on solubility and availability of mineral nutrients especially the micro nutrients. Sommers and Lindsay (1979) have shown that mobility and availability of metal ions is determined by soil pH. Similar observation was reported by Abebe et al. (2017) on influence of pH on Fe and Mn concentration in popcorn kernel. Our observation is similar to that of Ijarotimi et al. (2012) and Abebe et al. (2017).

The uptake concentration (mg/kg) of the minerals was in the order K^+^ > HPO_4_^2−^ > Mg^+2^ > Ca^2+^ > Fe^2+^ > Cr^6+^ > Zn^2+^ > Mn^2+^ > Cu^2+^. The availability of diverse minerals at different concentrations suggests that popcorn has enormous nutritional benefits. The nutritive value in the popcorn kernel is comparable to that of corn (Gopalan et al., 2012; Shahidi, 2015; Rouf Shah, 2016). The concentration of minerals such as Ca^2+^, Mg^+2^, K^+^, Cu^2+^, Fe^2+^ and Zn^2+^in popcorn kernel obtained from field supplied with higher rate of MSWC is within the acceptable limit and nutritional value of corn as reported by previous researchers (Mishra et al., 2006; Puga, 2013; Vaswani et al., 2016).

### Pattern of very toxic heavy metal uptake in kernels of popcorn

**Fig. 2:**
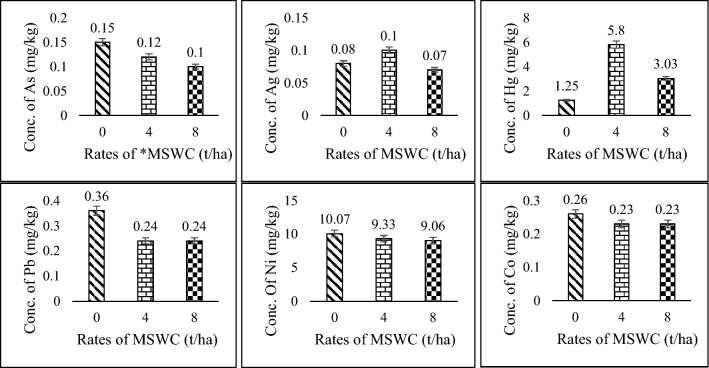
Uptake concentration (mg/kg) of some very toxic heavy metals in popcorn kernel. *MSWC = Municipal Solid Waste Compost, error bar represents LSD at *p* ≤ 0.05

The pattern of very toxic heavy metals uptake concentration in kernels of popcorn showed an inverse response with respect to the quantity of MSWC applied (Fig. 1). The concentration of the heavy metals in the kernel was in the order Ni^2+^ > Hg^2+^ > Pb^2+^ > Co^2+^ > As^3+^ > Ag^+^ in the control plots. Although, concentration of many of the heavy metals were highest in kernels obtained from unfertilized plots however, the concentrations of Ag^+^ and Hg^2+^ were highest in kernels harvested from plots supplied with 4 t/ha MSWC (Fig. 2). Concentration of Hg ion in corn kernel could bioaccumulate and lead to undesirable toxicological as well as ecological influence. Similar view is shared by Hu et al. (2016).

It is obvious from this trial that application of higher quantity or rate of MSWC reduced concentration of heavy metals translocated to kernels of popcorn. This could possibly be because the organic matter content (OMC) in the MSWC formed a complex of sort with the heavy metals thereby making them less available for uptake. The major component in the applied MSWC was OMC which probably formed organic acid such as humic acid that are known for their chelating ability. Tewari et al. (2018) reported that formation of metal ion complex with OMC impeded availability and uptake of heavy metals in polluted soils. It therefore means that the rate of mobility of heavy metal into grain of popcorn may have direct relationship with the concentration of the organic matter content. In which case, at higher OMC, availability and mobility of heavy metals into grains may be hindered. It is most doubtless that the OMC acted as a chelator according to Sekhon (2003) whereby, the metal(loid) pollutants were fixed to other complexes thus, seemingly unavailable for uptake and translocation.

Pattern of metal uptake also differs among the different ions. This may be linked to the activities of some of these metals relative to the constituent of other metabolites in the process of assimilate synthesis. For instance, the order in which Co and Ni ions were greatly assimilated compared to other hazardous metals assimilated implies that crop could decipher which ions were beneficial and which ones were toxic. This could explain why Co and Ni ions which are implicated as co-factors in enzymatic reactions and gene expression were among the minerals taken up better by this crop. Martínez-Ballesta et al. (2010a) have shown that these elements are classified as essential for both plant and human health and therefore, plant preference for them than the toxic ones. Nevertheless, their concentrations in the kernel appeared higher than required for normal plant growth as suggested by Szefer and Grembecka (2006) and Martínez-Ballesta et al. (2011).

In another view, Abebe et al. (2017) linked the concentration of metal(loid)s in agricultural product to plants propensity to absorb metals, solubility, abundance and mobility of the metal(loid)s in the soil. It is clear from this trial that the soil of the field was contaminated with heavy metals. This was evidence from the results of pre-trial soil analysis. The sources of these metal(loid) pollutants could be from frequent use of agrochemicals such as diverse forms of fossil fertilizers, herbicides, heavy metal-based insecticides. Apart from the synthetic origin, attempt to improve fertility of the arable field with sewage sludge, municipal solid waste and compost; could form other sources of the metal pollutants. This may have negative influence on the safety and quality of food products available for human consumption from the polluted rhizosphere. Similar observations had been reported by Abebe et al. (2017) on popcorn and cornflakes.

The different metal(loid)s detected in the kernel of popcorn grown on sandy loam Ferric Luvisol amended with MSWC is presented in Table 6. The concentration of the metalloids followed the pattern observed for heavy metals and plant nutrients. Highest concentration (mg/kg) of the different metalloids was obtained in unamended field while the least was observed in the plots supplied with 8 t/ha MSWC. The order of concentration was Al > Rb > Sb > Sr > Ba > Se > Pd ions in the unamended plots and Al > Rb > Sb > Ba > Sr > Se > Pd in the amended plots. Uptake concentration of Al and Pd ions were significantly higher in the unamended plots relative to uptake concentration recorded in plots fertilized with different quantities of MSWC. Uptake concentration of metalloids like Se, Rb, Sr Sb and Ba were not significantly affected by quantity of MSWC applied.

**Table 6:** Uptake concentration (mg/kg) of some potentially toxic elements in popcorn kernel

MSWC rate (t/ha)	Al	Se	Rb	Sr	Sb	Ba	Pd
0 (Control)	19.43	0.34	2.51	0.55	0.75	0.47	0.16
4	13.40	0.33	2.27	0.33	0.35	0.25	0.09
8	12.53	0.23	1.96	0.27	0.40	0.33	0.08
Mean	15.12	0.30	2.24	0.38	0.50	0.35	0.11
SEM ( ±)	1.13	0.02	0.19	0.04	0.10	0.04	0.01
LSD (*p* ≤ 0.05)	2.65	0.11	1.00	0.10	0.63	163.00	0.01

*MSWC* Municipal solid waste compost, *SEM* Standard error of means, *LSD* Least significant difference at *p* ≤ 0.05 mean (± standard error)

The concentration of these metalloids or non-essential elements in kernel decreased with increasing rate of MSWC. Based on our literature search, this is the first work where metalloids concentration in popcorn kernels is reported. Although, many of these metalloids are non-essential to plant development, but some, like Se had been reported by Peereboom (1985), Fraga (2005) to function as an important component of human cell membrane enzyme. Report of Iskander and Davis (1992) has shown presence of some trace elements like Rb, Sb, Se in bread. Also, Gunes et al. (2008) have reported variation in uptake of Ti, Rb, Ba and Si ions in silage corn.

The concentration of essential mineral nutrient in the kernels decreased significantly with increase in quantity of MSWC applied and highest in the unamended plots (Table 7). The kernel was richer in P, K and Mg compared to other mineral nutrients. Equally, the concentrations of Cr, Zn and Ca were appreciably higher in popcorn kernels relative to Mn, Mo and Cu concentrations.

**Table 7:** Bio-concentration (mg/kg) of essential plant nutrients in popcorn kernel

MSWC rate (t/ha)	K	P	Mg	Ca	Cu	Fe	Mn	Mo	Zn
Control (0)	353.40	2282.89	233.56	1.94	0.07	0.13	0.01	0.03	2.68
4	291.71	1642.54	163.51	1.21	0.05	0.10	0.01	0.03	1.69
8	251.15	1319.34	135.78	0.87	0.05	0.09	0.01	0.03	1.32
Mean	298.75	1748.26	177.62	1.34	0.06	0.10	0.01	0.03	1.89
SEM ( ±)	5.02	76.09	10.28	0.09	0.003	0.073	3.26 × 10^–4^	9.45 × 10^–5^	0.15
LSD (*p* < 0.05)	22.581	342.46	46.27	0.43	1.407	3.266	1.64 × 10^–3^	4.26 × 10^–4^	0.69
*F* value	0.002**	0.007**	0.014*	0.009**	0.019*	0.046*	0.04*	0.033*	0.017*

*MSWC* Municipal solid waste compost, *SEM* Standard error of means, *LSD* Least significant difference at *p* ≤ 0.05

*Significant at 0.01

**Significant at 0.001

### Bioavailability minerals and transfer coefficient of some toxic heavy metals

The bioavailability of the essential plant nutrients in popcorn kernels attested to the fact that popcorn is a rich source of these mineral nutrients which are precursors of many bioactive compounds, vitamins and minerals, necessary in human diets and nutrition. The higher bioavailability of K, P and Mg ions in the kernels agrees with the findings of Martínez-Ballesta et al. (2010b) on nuts and seeds. Also, Szefer and Grembecka (2006) and Concepts (2006) have also reported that P and Mg ions recommended daily intake ranged between 3500 mg and 200–400 mg/day, respectively. This goes to say that the kernel is indeed a good source of healthy diet. Other trace element such as Cu has concentrations range which was higher than those reported for vegetable and fruits (Szefer & Grembecka, 2006).

Bio-concentration or transfer coefficient of some toxic heavy metals and metalloid in popcorn kernels is shown in Table 8. Higher bioavailability of very toxic heavy metals was recorded in the control plots while least concentration was observed in plots supplied with 8 t/ha municipal solid waste compost. Kernels harvested from unfertilized field had highest bio-concentration of potentially toxic elements relative to the kernels obtained from plots fertilized with different rates of MSWC.

**Table 8:** Bio-concentration (mg/kg) of some potentially toxic elements in popcorn kernel

MSWC Rate (t/ha)	As	Pb	Ni	Co	Al	Cr	Cd
Control (0)	0.19	9.97 × 10^–8^	10.64	0.21	1.19	4.18	ND
4	0.15	7.25 × 10^–8^	9.51	0.20	0.89	3.88	ND
8	0.11	6.25 × 10^–8^	8.94	0.19	0.74	3.34	ND
Mean	0.15	7.82 × 10^–8^	9.70	0.20	0.94	3.80	ND
LSD (*p* < 0.05)	2.08 × 10^–4^	1.89 × 10^–8^	0.59	1.189 × 10^–2^	0.174	0.256	–
*F*-value	1.01 × 10^–7^**	0.017*	0.006**	0.017*	0.008**	0.004**	–
FAO/WHO (mg/kg)	0.20	0.20	2.70	0.48	1.00	2.30	0.20

*MSWC* Municipal solid waste compost, *LSD* Least significant difference at *p* ≤ 0.05, *ND* not detected

*Significant at 0.01

**Significant at 0.001

***Significant at 0.0001

Generally, bioavailability of Ni and Al in the unfertilized plots were higher than recommended standard given by FAO/WHO (Commission, 2011; Sharma, 2013). Chromium concentration in the kernel decreased with increasing rate of MSWC and beyond the acceptable limit. Many of the hazardous heavy metals concentration in the kernels obtained from field amended with MSWC were within the minimum standard allowed for corn by FAO/WHO guidelines. It therefore suggests that the concentrations of Ni and Cr which were beyond the FAO/WHO standards may portend health danger to consumers of corn harvested from field fertilized with this amendment. Hence, growers should be courteous when applying MSWC rich in Ni or Cr ions to amend nutrient deficient soils since, the uptake and bio-concentration of such metals could attain dangerous threshold to human health. Nickel is one of the essential mineral nutrients required for urease synthesis but at high bioavailability in plant tissue, it could cause human health conditions. Similar observation had been reported by Marschner (2011) on higher plants. In the same vein, accumulation of Cr beyond the acceptable limit may become hazardous to humans and livestock. According to Oliveira (2012), Cr contamination has increased because of its divers anthropogenic use which has resulted in its bioaccumulation in the environment. On the other hands, the lower bioaccumulation of other toxic heavy metals in kernel of popcorn implies that despite high concentration of these metals in the compost especially Pb, MSWC is still a vital source for improving fertility of sandy loam soil in semi-arid region.

### Average daily metal intake

The average daily intake (ADI) determined using oral intake of popcorn kernels by adults and children is presented in Table 9. The ADI of all the potentially toxic elements were below the recommended daily intake. Although, the intake was higher in the kernels from unamended field relative to other plots supplied with different rates of MSWC. Application of MSWC to improve popcorn performance significantly influenced heavy metal intake. Popcorn grains harvested from unfertilized plots had highest ADI of the potentially toxic elements which were significantly higher than metal intake in kernels/grains from amended plots. The ADI of the potentially toxic elements in the kernels is in the order 8t/ha < 4t/ha < 0 t/ha.

**Table 9:** Average daily intakes of potentially toxic elements in popcorn kernel (mg/kg/day)

MSWC rate (t/ha)	Pb		Hg		As		Zn		Mo	
	Adult	Child	Adult	Child	Adult	Child	Adult	Child	Adult	Child
Control	2.13E−07	1.99E−06	7.32E−07	6.83E−06	8.86E−08	8.27E−07	1.86E−05	1.74E−04	6.21E−07	5.79E−06
4	1.41E−07	1.31E−06	3.41E−06	3.18E−05	6.86E−08	6.40E−07	1.13E−05	1.06E−04	6.20E−07	5.79E−06
8	1.39E−07	9.76E−07	1.78E−06	9.19E−06	5.66E−08	4.40E−07	1.01E−05	6.74E−05	6.10E−07	4.29E−06
SD( ±)	0.21	na	1.00	0.03	5 × 10^–3^	5 × 10^–3^	11.00	8.00	0.05	3 × 10^–3^
RDI^++^	4.67E−09	2.92E−07	4.95E−07	4.85E−06	7.34E−09	1.36E−07	1.34E−06	2.49E−05	8.03E−08	1.69E−06
LSD *p* < 0.05):	1.30E−08	8.11E−07	1.37E−06	1.35E−05	2.04E−08	3.76E−07	3.71E−06	1.15E−04	2.23E−07	4.68E−06
*p*	**	ns	*	*	**	ns	ns	*	**	ns

MSWC rate (t/ha)	Cu		Ni		Co		Fe		Cr	
	Adult	Child	Adult	Child	Adult	Child	Adult	Child	Adult	Child
Control	2.31E−06	2.15E−05	5.91E−06	5.52E−05	1.50E−07	1.40E−06	9.11E−05	8.50E−04	2.81E−05	2.62E−04
4	1.51E−06	1.41E−05	5.48E−06	5.11E−05	1.37E−07	1.28E−06	5.87E−05	5.48E−04	2.65E−05	2.48E−04
8	1.54E−06	1.08E−05	5.32E−06	3.55E−05	1.37E−07	9.28E−07	6.59E−05	4.47E−04	2.25E−05	1.68E−04
SD( ±)	0.90	0.70	0.002	0.01	na	na	16.30	20.50	0.0035	0.002
RDI^++^	1.31E−07	2.27E−06	1.70E−07	1.01E−05	2.45E−09	2.68E−07	4.47E−06	1.41E−04	1.91E−06	5.16E−05
LSD (p < 0.05)	3.63E−07	8.93E−06	7.81E−07	2.81E−05	6.80E−09	7.43E−07	1.24E−05	3.92E−04	5.29E−06	1.43E−04
*p*	**	ns	ns	ns	**	ns	**	ns	ns	ns

*MSWC* Municipal Solid Waste Compost, *LSD* Least Significant Difference at p ≤ 0.05, *SD* Standard deviation

**Significant at *p* = 0.001, *significant at *p* = 0.05

++Recommended daily dietary allowance (mg/day /person) (Organization, 2018)

The implication of this is that, the applied MSWC appeared to reduce bioaccumulation of potentially toxic metals in kernels of popcorn. This suggests that the applied MSWC possibly remediated the metal pollutants present in the soil. Compost has been shown to remediate site polluted with heavy metals via anthropogenic or agronomic practices like application of agrochemicals for enhancing the performance of cultivated crops (Lim et al., 2016). We share similar view with Rusmini et al. (2018) who reported that addition of compost increased the number of soil microbes with significant reduction in heavy metals than in the soil without compost.

### Health risk of the transferred heavy metals from compost to popcorn kernels

The health risk of the transferred heavy metals is presented in Table 10. The health risk index varied significantly with respect to the rates of MSWC applied. The health risk was significantly inconsequential in kernel obtained from field amended with higher quantity of the compost while the unamended plots showed appreciable risk index. Generally, the risk index showed that the metals transferred do not constitute health risk to both adult and children population.

**Table 10:** Health Risk index of potentially toxic elements in popcorn kernels

MSWC rate (t/ha)	As		Cr		Cu		Fe		Hg	
	Adult	Children	Adult	Children	Adult	Children	Adult	Children	Adult	Children
Control	8.27E−04	3.31E−04	5.62E−03	1.05E−02	6.92E−05	1.29E−04	2.73E−03	1.46E−01	2.28E−04	2.28E−04
4	6.40E−04	2.56E−04	5.30E−03	9.90E−03	4.54E−05	8.48E−05	1.76E−03	9.40E−02	1.06E−03	1.06E−03
8	5.28E−04	2.11E−04	4.50E−03	8.41E−03	4.62E−05	8.62E−05	1.98E−03	1.05E−01	5.53E−04	5.53E−04
SD( ±)	8.81E−05	3.52E−05	4.53E−04	8.46E−04	5.51E−06	1.03E−05	1.47E−04	7.82E−03	2.09E−04	2.09E−04
LSD (*p* < 0.05)	2.16E−04	8.62E−05	1.11E−03	2.07E−03	1.35E−05	2.52E−05	3.59E−04	1.91E−02	5.11E−04	5.11E−04
Sig	*	*	ns	ns	**	**	**	**	*	*

MSWC rate (t/ha)	Mn		Ni		Pb		Co		Zn	
	Adult	Children	Adult	Children	Adult	Children	AdultAdult	Children	Adult	Children
Control	2.45E−04	4.57E−04	1.18E−05	2.21E−05	3.56E−05	6.64E−05	2.43E−06	4.54E−06	5.58E−04	1.04E−03
4	1.66E−04	3.10E−04	1.10E−05	2.04E−05	2.35E−05	4.38E−05	2.22E−06	4.14E−06	3.40E−04	6.35E−04
8	1.81E−04	3.37E−04	1.06E−05	1.99E−05	2.31E−05	4.32E−05	2.22E−06	4.14E−06	3.02E−04	5.63E−04
SD( ±)	3.21E−05	2.45E−05	4.14E−07	7.74E−07	8.46E−07	1.58E−06	4.96E−08	9.26E−08	4.13E−05	7.71E−05
LSD (p < 0.05)	3.21E−05	5.99E−05	1.01E−06	1.89E−06	2.07E−06	3.86E−06	1.21E−07	2.27E−07	1.01E−04	1.89E−04
p	**	**	ns	ns	**	**	**	**	**	**

*MSWC* Municipal solid waste compost, *LSD* Least significant difference at *p* ≤ 0.05, *SD* standard deviation

**Significant at *p* = 0.001, *significant at *p* = 0.05

Our findings in this study showed that application of MSWC could serve dual purpose of improving soil fertility and remediating fields that were contaminated with potentially toxic metal pollutants such as Pb, As, Al and others. Abou Jaoude et al. (2019) have also shown that MSWC contains SOM that inextricably adsorbed metalloids and heavy metals in polluted field and also capable of reducing toxicological effect on soil microbiota. This might contribute to minimizing mobilization and accumulation of potentially toxic elements into food chain beyond the threshold level that constitute hazards to human and livestock (Yaylalı-Abanuz, 2011).

The HQ and HI values were less than one (< 1) for adults at all rates of MSWC although, the HQ and HI were higher in plots supplied with 4 t/ha (Table 10). Obviously, the heavy metals intake would not constitute health risk to the adult population. The evaluated HQ value for children was less than 1 (< 1) for kernels obtained from unamended plots and field amended with 8 t/ha MSWC. However, kernel obtained from plots supplied with 4 t/ha MSWC had HQ value that was greater than one (> 1). This implies that children who consume popcorn kernels harvested from plots amended with 4 t/ha are in danger of high non-cancer health risk. Suboptimal MSWC quantity may not possess sufficient microbes or organic matter that could help immobilize some the potentially toxic elements that may induce health risk. It is therefore important that growers take cognizance and equilibrate the quantity and chemical constituents of MSWC such that the amount applied to their fields would not pose health risk when used as soil amendment.

The high HQ observed in plots supplied 4 t/ha MSWC was contributed by Cr and Hg ions to give a total of 1.23. Generally, Cr and Hg ions contributed high values to the observed HQ of both adult and children. Kamunda et al. (2016) have reported that Cr ion in soils from Witwatersrand Gold Mining Basin had HQ which was 43 times higher than the South African maximum allowable limit and some other countries. Application of higher rate of MSWC above 4 t/ha would suffice for improving fertility of sandy loam soil, growth and yield of popcorn without causing any health risk. Similar report was recorded by Yaylalı-Abanuz (2011) in capsicum, okra, cowpea and eggplant grown on vicinity of a tailing pond in China.

## Conclusion

Compost is a good source of plant mineral nutrients but could also serve as source of pollutants in agricultural field. Amendment of sandy loam soil with Municipal Solid Waste Compost (MSWC) alleviated the poor-quality status of sandy loam Ferric Luvisol. Popcorn grown on the low nutrient deficient soil amended with MSWC had superior performance relative to the unamended soil. The applied MSWC at 8 t/ha demonstrated potential for remediating potentially toxic elements such as Cr, As, Ni, Pb, Cd and Hg without any deleterious effect on the kernels. The uptake concentration and bioavailability of these elements in popcorn kernels were within the permissible threshold that will not constitute any health risk to both adult and children population (Table 11).

**Table 11:** Hazard quotient for potentially toxic element in adults and children based on oral exposure

	Adult			Children		
	0 t/ha	4 t/ha	8 t/ha	0 t/ha	4 t/ha	8 t/ha
Cr	2.81E−01	2.65E−01	2.25E−01	5E−01	5E−01	3E−01
As	2.55E−02	7.86E−03	6.41E−03	5E−02	1E−02	9E−03
Hg	7.32E−02	3.41E−01	1.78E−01	1E−01	6E−01	2E−01
Pb	1.78E−03	1.17E−03	1.16E−03	3E−03	2E−03	2E−03
Fe	1.68E−04	1.08E−04	1.21E−04	7E−07	5E−07	4E−07
Ni	8.87E−03	8.21E−03	7.98E−03	2E−02	2E−02	1E−02
Cu	1.87E−03	1.23E−03	1.25E−03	3E−03	2E−03	2E−03
Mo	3.72E−04	3.72E−04	3.66E−04	7E−04	7E−04	5E−04
Zn	1.86E−03	1.13E−03	1.01E−03	3E−03	2E−03	1E−03
HQ	3.95E−01	6.26E−01	4.21E−01	7.37E−01	1.17E + 00	5.44E−01
